# A Comparison of Inbreeding Depression in Tropical and Widespread *Drosophila* Species

**DOI:** 10.1371/journal.pone.0051176

**Published:** 2013-02-27

**Authors:** Jesper S. Bechsgaard, Ary A. Hoffmann, Carla Sgró, Volker Loeschcke, Trine Bilde, Torsten N. Kristensen

**Affiliations:** 1 Department of Bioscience, Aarhus University, Aarhus, Denmark; 2 Department of Genetics and Bio21 Institute, Melbourne University, Melbourne, Australia; 3 School of Biological Sciences, Monash University, Melbourne, Australia; 4 Department of Molecular Biology and Genetics, Aarhus University, Tjele, Denmark; 5 NordGen - Nordic Genetic Resource Center, Ås, Norway; Ludwig-Maximilians-Universität München, Germany

## Abstract

The evolutionary history of widespread and specialized species is likely to cause a different genetic architecture of key ecological traits in the two species groups. This may affect how these two groups respond to inbreeding. Here we investigate inbreeding effects in traits related to performance in 5 widespread and 5 tropical restricted species of *Drosophila* with the aim of testing whether the two species groups suffered differently from inbreeding depression. The traits investigated were egg-to-adult viability, developmental time and resistance to heat, cold and desiccation. Our results showed that levels of inbreeding depression were species and trait specific and did not differ between the species groups for stress resistance traits. However, for the life history traits developmental time and egg-to adult viability, more inbreeding depression was observed in the tropical species. The results reported suggest that for life history traits tropical species of *Drosophila* will suffer more from inbreeding depression than widespread species in case of increases in the rate of inbreeding e.g. due to declines in population sizes.

## Introduction

A species' genetic architecture, referring to the number of segregating functional variants, including dominance and epistatic interactions, underlying phenotypic traits, is formed by phylogenetic, demographic and ecological processes, and determines its biological performance, such as its basal resistance to environmental stressors and the ability to adapt evolutionary and through adaptive phenotypic plasticity to environmental changes [1]. The genetic architecture of a population or species also dictates how it will cope with inbreeding since the cost of inbreeding is under the influence of allele frequencies, amount of dominance variance and the level of inbreeding [2].

Some *Drosophila* species are restricted to narrow habitat ranges in the tropics (‘tropical species’), whereas others occupy habitats ranging from tropic to temperate areas (‘widespread species’). Widespread species are exposed to different, more variable and diverse ecological conditions compared to specialized restricted tropical species, which will likely lead to different selection pressures and subsequent genetic divergence of the two species groups through evolutionary adaptation [3]–[6]. In addition tropical species of *Drosophila* are on average more closely related to each other than to widespread species [6] adding a phylogenetic component to the differences among the two species groups. Widespread and tropical restricted species of *Drosophila* are for those reasons likely to have a different genetic architecture, especially in key ecological traits involved in environmental stress resistance.

Tropically restricted *Drosophila* species have recently been hypothesized to be more prone to experience reduced population sizes and extinction as compared to more widespread *Drosophila* species as the environment changes. This is based on evidence from a number of studies showing that widespread species have higher levels of basal resistance to climatic extremes (temperature and humidity) when compared to tropical climate specialists [6]–[9], and that some tropical restricted species of *Drosophila* have lower adaptive evolutionary potential in the ecological key traits desiccation and cold resistance [10], [11]. Demographic effects like bottlenecks, inbreeding and/or genetic drift seem to be excluded as causing these differences since levels of neutral genetic variation seem to be similar among the two species groups [11], [12] suggesting that historical (phylogenetic) and current (ecological) selection pressures are responsible [6], [9].

Based on the hypothesis that tropical and widespread species of *Drosophila* have a different genetic architecture, it is of interest to investigate if the two species groups respond differently to inbreeding. If the cost of inbreeding differs between the species groups, this may influence the extinction probabilities of these species groups when they experience an increase in inbreeding rates in their natural habitats, due to for instance reductions in population size as may occur under future climate changes. Here, we study the consequences of inbreeding in 5 widespread and 5 tropical restricted species of *Drosophila* by investigating inbred and outbred lines for the traits egg-to-adult viability, developmental time, and resistance to cold, heat and desiccation stresses. Generally, we found strong line, species and trait specific effects of inbreeding. Tropical species on average suffered more from inbreeding depression compared to widespread species for life history traits, whereas no differences among species groups were observed in stress resistance traits.

## Materials and Methods

### Collection of flies

A population from each of 10 *Drosophila* species was collected during 2007 and 2008 (December to April) in North Queensland, along the Australian east coast. Five of the species are restricted to the tropics, mostly rainforest habitats (*D. birchii*, *D. bunnanda*, *D. bipectinata*, *D. sulfurigaster* and *D. pseudoananassae*), while the other 5 are cosmopolitan or widespread species whose habitat includes temperate regions as well (*D. melanogaster*, *D. simulans*, *D. hydei*, *D. repleta* and *D. serrata*). Average latitude of the tropical and widespread species investigated here range from 13.04° to 18.15° and 101.75° to 129.40°, respectively (based on registrations entered in the taxodros database available at http://www.taxodros.uzh.ch). For each species, 18 to 22 inseminated females from a single population were caught and brought to the laboratory to establish mass bred populations, which were maintained for 15 generations with at least 1000 individuals before outbred and inbred lines were established. The expected loss of heterozygosity during this process is calculated according to [13] and constitutes about 3%. Flies were reared on an oat-sugar-yeast-agar medium under a 12∶12 h light/dark cycle at 20°C prior to performing the experiments. Details regarding sampling locations are described in [8]. No permits were required for the described field collections.

### Breeding regimes

From each species, 3 outbred control lines and 20 inbred lines were established from the mass bred population. The outbred lines were started from about 750 individuals, whereas the inbred lines were started from single pairs of virgin females and males which were subsequently run through four generations of full sib mating, resulting in an expected inbreeding level of 0.59 (F_t_ = 1/4(1+2F_t−1_+F_t−2_)). Thereafter inbred lines were expanded to about 500 individuals before performing the experiments. Between 3 and 10 inbred lines per species were used for experiments (Table S1). For species where more than 10 inbred lines were available 10 randomly collected lines were used (11, 13 and 14 lines were available to select from from *D. sulfurigaster*, *D. repleta* and *D. pseudoananassae*, respectively). The experiments were done in the second generation after the last inbreeding round (see [14] for more details).

### Egg collection

From each inbred and control line 20 eggs were collected into each of 10 vials with 7 ml medium, and incubated at 20°C. Flies emerging from these eggs were used for assessing stress resistance traits (see below). From prior knowledge of generation times, collection of eggs was timed so that flies from all species emerged over as short a time span as possible. Flies used for experiments were all between 3 and 8 days old.

### Developmental time and egg-to-adult viability

Ten vials (with 7 ml medium) per line each containing 20 eggs were set up as specified above. Emerging flies were counted twice a day (at 8:00 and 20:00 h). Egg-to-adult viability was scored as the number of males and females emerging from each vial and developmental time estimated as the time taken to develop from an egg to an adult female or male fly. Flies were all reared at 20°C degrees since this temperature is considered non-stressful for all species investigated. Emerging flies were used for the temperature and desiccation assays described below.

### Temperature resistance assays

1. Heat knock down resistance. Ten flies per line and sex were individually put into 5 ml glass vials and submerged into a preheated 38°C water bath. Heat resistance was scored as the time until the flies were unable to move any of their body parts.
2. Chill coma recovery time. Ten flies per line and sex were individually put into 5 ml glass vials and submerged into a 0°C water bath and kept there for 3 hours. The vials were then put at room temperature, and chill coma recovery was scored as the time when the flies were able to stand on their legs.
3. Critical thermal minimum (CTmin). Ten flies per line and sex were individually put into 5 ml glass vials and submerged into a 20°C water bath. The temperature was then decreased by 0.1°C per minute. CTmin was scored as the temperature at which flies were in coma unable to move any body parts (see [14] and [15] for details regarding this assay).

### Desiccation resistance assay

Ten flies per line and sex were individually put into 5 ml glass vials covered with gauze. Vials were then put into sealed aquariums. Prior to setting up the experiment, desiccant was added to the aquariums to reach air humidity of 0–5%. Desiccation resistance was scored as the time until the flies were unable to move any of their body parts. Vials were checked every hour.

### Statistics

For each trait we used a nested ANOVA to test for effects of line (nested within species and breeding regime, as random effects), species, breeding regime (inbred or outbred), sex and the interactions between the fixed effects. Inbreeding depression (δ) was estimated for each trait using the formula δ = 1 – mean inbred/mean control [16]. For traits where individuals with lower values are expected to be more fit(develommental time, CTmin and chill coma recovery time) inbreeding depression was estimated using the formula δ = 1 – mean control/mean inbred. Mean control was the average of the three control lines and each inbred line was standardized to this value for all traits in all species. Positive values were indicative of inbreeding depression for all traits. . Effects of species, sex and their interaction on the level of inbreeding depression in each trait were tested with ANOVAs. Data were generally normally distributed although deviations from normality were observed in a few cases. Egg-to-adult viability data were arc sin square root transformed to improve normality of the data. For the other traits analyses were performed on non-transformed data. Differences between levels of inbreeding depression between widespread and tropical species were tested with a non-parametric Mann-Whitney test for life history traits (developmental time and egg-to-adult viability) and stress resistance traits (CTmin, cold recovery time, heat knock down time and desiccation resistance), respectively. For each species within the two species groups, estimates of inbreeding depression were averaged across males and females for each line. Thus for each trait and species we had an estimate of inbreeding depression for each line. These data were used to test for effects of species group using an ANOVA with species nested within distribution (tropical or widespread species group).

## Results

**Figure 1:**
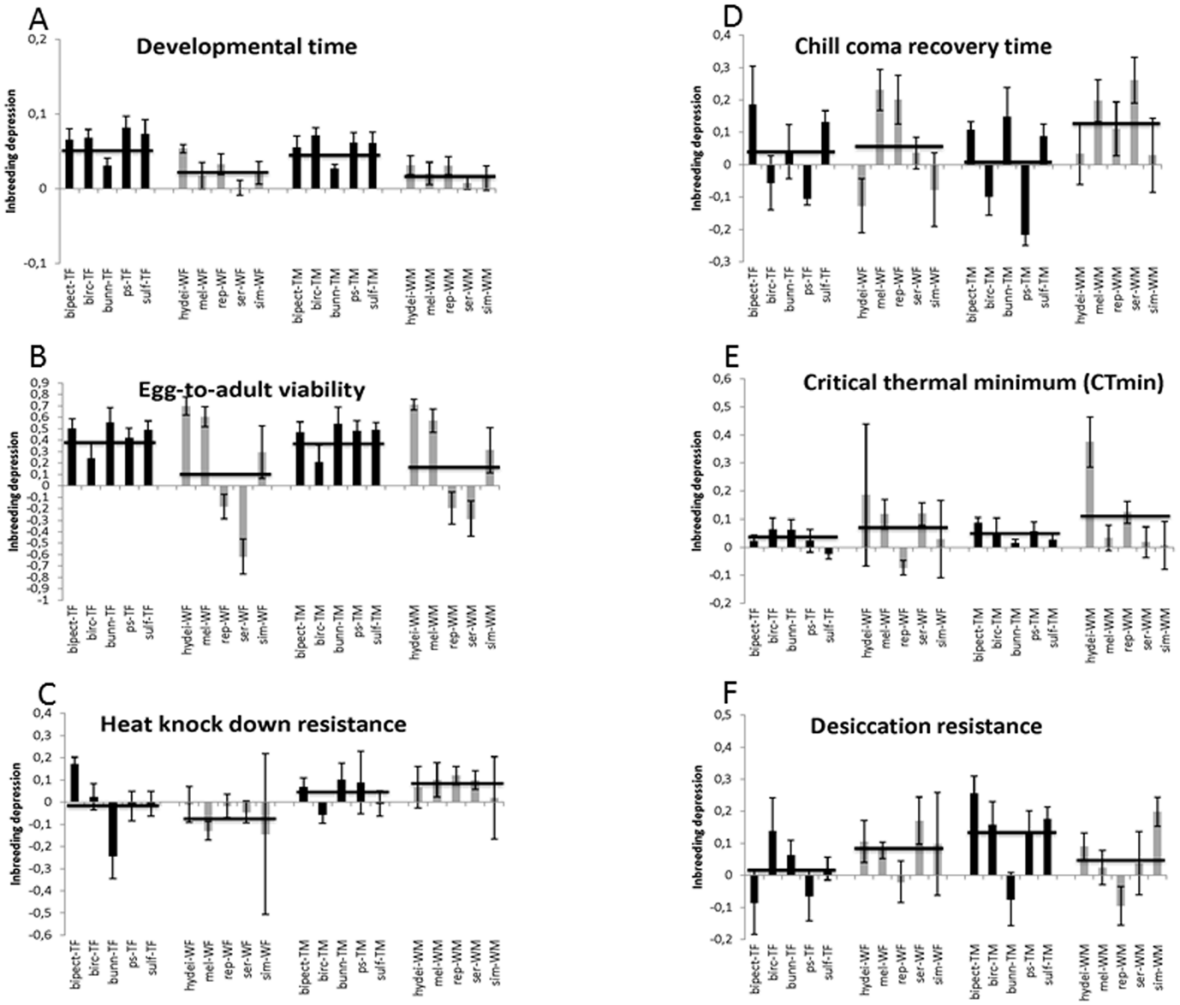
Inbreeding depression for each of 6 traits (± SE) (a: ‘Developmental time’, b: ‘Egg-to-adult viability’, c: ‘Heat knock down resistance’, d: ‘Chill coma recovery time’, e: ‘Critical thermal minimum (CTmin)’, f: ‘Desiccation resistance’). Data are split into 4 groups: tropical females (TF), tropical males (TM), widespread females (WF) and widespread males (WM). Horisontal lines represent averages for each of the 4 groups (TF, TM, WF, WM). The species are: *D. bipectinata* (bipect), *D. birchii* (birc), *D. bunnanda* (bunn), *D. hydei* (hydei), *D. melanogaster* (mel), *D. pseudoananassae* (ps), *D. repleta* (rep), *D. serrata* (ser), *D. simulans* (sim) and *D. sulfurigaster* (sulf). See ‘Materials and methods’ for a description of each assay.

Developmental time: Line (nested within species and breeding regime), species, breeding regime (inbred or outbred), sex and the species×sex interaction all significantly affected developmental time (line: F_79,1767_ = 17.47, P<0.001; species: F_9,1767_ = 207.95, P<0.001; breeding regime: F_1,1767_ = 33.52, P<0.001; sex: F_1,1767_ = 231.53, P<0.001; species×sex: F_9,1767_ = 5.65, P<0.001; Fig. 1a, Table S1). Remaining factors in the analysis were non-significant (results not shown). Inbreeding on average increased developmental time by 3.8 and 4.5% across species in males and females, respectively (Fig. 1a). The level of inbreeding depression (faster developmental time is interpreted as beneficial) differed between species (F_9,118_ = 6.09, P<0.001, Fig. 1), whereas it was similar for males and females of the same species (F_1,118_ = 0.80, NS). Levels of inbreeding depression were also not affected by the species×sex interaction (F_9,118_ = 0.22, NS).

Egg-to-adult viability: Line, species, breeding regime (inbred or outbred) and the species×breeding regime interaction all significantly affected egg-to-adult viability (line: F_80,1862_ = 12.31, P<0.001; species: F_9,1862_ = 2.25, P<0.05; breeding regime: F_1,1862_ = 37.68, P<0.001; species×breeding regime F_9,1862_ = 3.46, P<0.01; Fig. 1b, Table S1). Other factors in the model were non-significant (results not shown). Inbreeding on average decreased viability by 33.1 and 30.0% across species in males and females respectively (Fig. 1b). The level of inbreeding depression differed between species (F_9,120_ = 21.29, P<0.001, Fig. 1). Neither sex (F_1,120_ = 0.26, NS) nor the interaction species×sex impacted on the level of inbreeding depression (F_9,120_ = 0.52, NS).

Heat knock down resistance: Line (nested within species and breeding regime), species, and the breeding regime×sex and species×sex interactions significantly affected heat knock down resistance (line: F_69,1512_ = 1.93, P<0.001; species: F_9,1512_ = 69.90, P<0.001; breeding regime×sex F_9,1512_ = 7.73, P<0.01; species×sex: F_9,1512_ = 1.96, P<0.05; Fig. 1c, Table S1). Breeding regime, sex and the remaining interactions between the main effects did not affect heat knock down resistance (results not shown). Heat knock down time was on average 4.2% and 6.0% lower in inbred compared to outbred flies (Fig. 1c). The level of inbreeding depression differed between males and females (F_1,85_ = 6.32, P<0.05). The impact of inbreeding was not affected by species (F_9,85_ = 0.67, NS) nor by the interaction species×sexes (F_9,85_ = 1.12, NS) (Fig. 1c).

Chill coma recovery time: Line (nested within species and breeding regime), sex, species and the species×breeding regime and species×sex interactions all significantly affected egg-to-adult viability in chill coma recovery time (line: F_77,1704_ = 1.97, P<0.001; sex: F_1,1704_ = 11.18, P<0.001; species: F_9,1704_ = 116.99, P<0.001; species×breeding regime F_9,1704_ = 3.38, P<0.01; species×sex: F_9,1704_ = 5.11, P<0.001; Fig. 1d, Table S1). Remaining factors in the model were non-significant (results not shown). On average inbreeding increased chill coma recovery time (decreased cold resistance) by 6.1 and 4.6% in males and females, respectively. The impact of inbreeding differed between species (F_9,108_ = 6.46, P<0.001, Fig. 1d). Neither sex (F_1,108_ = 0.36, NS) nor the interaction species×sex impacted on the level of inbreeding depression (F_9,108_ = 1.61, NS).

CTmin: Line (nested within species and breeding regime), sex, species and breeding regime all significantly affected CTmin (line: F_81,2063_ = 1.88, P<0.001; sex: F_1,2063_ = 6.66, P<0.01; species: F_9,2063_ = 149.60, P<0.001; breeding regime: F_1,2063_ = 6.84, P<0.05; Fig. 1e, Table S1). Remaining factors in the model were non-significant (results not shown). Inbred females and males had on average a CTmin that was respectively 5.3 and 8% higher (less cold resistant) compared to outbred females and males. The level of inbreeding depression differed between species (F_9,116_ = 3.00, P<0.01), whereas it was the same in males and females of the same species (F_1,116_ = 1.13, NS). The interaction species×sex was significant (F_9,116_ = 2.05, P<0.05) (Fig. 1e).

Desiccation resistance: Line (nested within species and breeding regime), sex, species, breeding regime (inbred or outbred) and the species×sex and the species×sex×breeding regime interactions all significantly affected desiccation resistance (line: F_74,1708_ = 6.06, P<0.001; sex: F_1,1708_ = 46.64, P<0.001; species: F_9,1708_ = 44.89, P<0.001; breeding regime: F_1,1708_ = 4.93, P<0.05; species×sex: F_9,1708_ = 34.30, P<0.001; species×sex×breeding regime: F_9,1708_ = 2.23, P<0.05; Fig. 1f, Table S1). The remaining factors in the model were non-significant (result not shown). Inbreeding on average decreased desiccation resistance by 9.0 and 5.1% across species in males and females respectively (Fig. 1f). The level of inbreeding depression did not differ between species (F_9,101_ = 1.73, NS) nor sexes (F_1,101_ = 1.17, NS), but was affected by the interaction species×sex (F_9,101_ = 2.03, P<0.05) (Fig. 1f).

### Inbreeding effects in widespread and tropical restricted species

For developmental time more inbreeding depression was observed in tropical compared to widespread species for both sexes (females: F_1,8_ = 9.71, P<0.05; males: F_1,8_ = 14.86, P<0.01, Fig. 1a). A non-significant trend in the same direction was observed for egg-to-adult viability (females: F_1,8_ = 1.22, P<0.10; males: F_1,8_ = 1.07, P<0.20, Fig. 1b). For the remaining traits, no suggestion of a difference in the impact of inbreeding on tropical and widespread species was evident (Fig. 1c–f).

**Figure 2:**
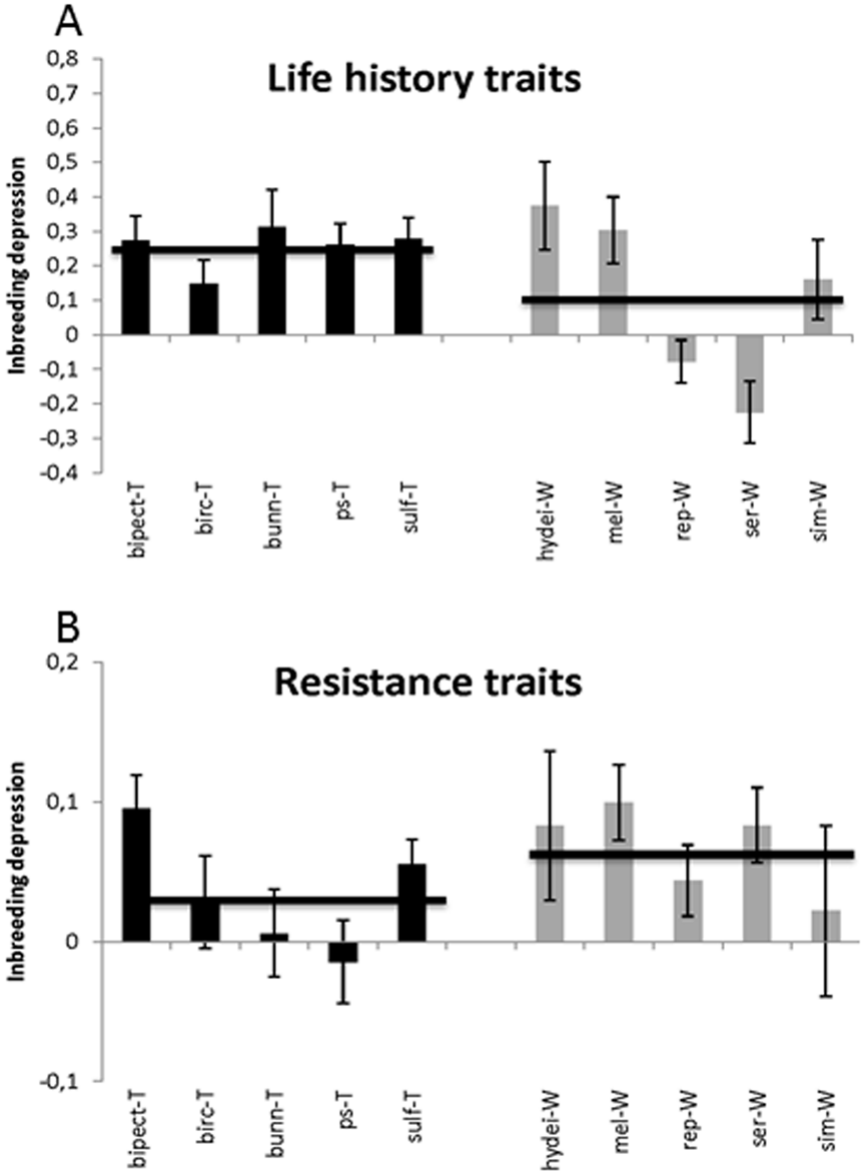
Average inbreeding depression for the 10 species based on a) average inbreeding depression of the two investigated life history traits for each species (± SE) and b) average inbreeding depression of the four investigated stress resistance traits for each species (± SE). Black bars are tropical species and grey bars are widespread species. Horizontal black lines represent averages for the tropical and widespread species. The species are: *D. bipectinata* (bipect), *D. birchii* (birc), *D. bunnanda* (bunn), *D. hydei* (hydei), *D. melanogaster* (mel), *D. pseudoananassae* (ps), *D. repleta* (rep), *D. serrata* (ser), *D. simulans* (sim) and *D. sulfurigaster* (sulf). Symbols: ‘T’ = ‘tropical’ and ‘W’ = ‘widespread’.

Splitting data into life history (developmental time and egg-to adult viability) and stress resistance traits, we found that tropical species suffered on average more from inbreeding depression in life history traits compared to widespread species (P<0.001; Fig. 2a) whereas no difference was observed for stress resistance traits (NS, Fig. 2b). When considering all traits together, there was no effect of species group on the level of inbreeding depression (NS).

## Discussion

In this study we investigated the effect of inbreeding in 5 tropical restricted and 5 widespread species of *Drosophila*. The two species groups have a different evolutionary history and they occupy different habitats and thus are likely to have experienced different selection pressures. We tested the hypothesis that, due to likely differences in the genetic architecture of the two species groups, the level of inbreeding depression differs between widespread and tropical species groups. For the stress resistance traits investigated we found no evidence to support this hypothesis (Fig. 2b), but for the life history traits we found that tropical species suffered more from inbreeding depression compared to widespread species, although large species differences were observed (Fig. 2a). The higher inbreeding depression in tropical species for life history traits is partly due to the fact that for two widespread species inbred flies have higher fitness compared to outbred flies (Fig. 1a,b and 2a). Purging of deleterious recessive alleles during the process of inbreeding could explain this result if the efficiency of purging were species specific. However, given the fast rate of inbreeding (full sib mating) used here purging is not likely to be efficient [17], [18]. Thus despite a large species effect and a relatively low number of species investigated the observation that inbreeding depression on average tend to be higher for life history traits in tropical compared to widespread species is likely caused by a distinct genetic architecture in the two species groups.

For cold and desiccation resistance, previous studies have provided evidence suggesting that the genetic architecture is indeed different between the two species groups for these traits [10], [11]. Tropical species have, in contrast to widespread species, very low heritability for cold and desiccation resistance [10], [11]. Two opposing hypotheses have been suggested to explain this result: 1) loci influencing those two traits have decayed (loss of function) in the tropical species [19], the reason being that they are never in use in a humid and warm habitat, and 2) loci influencing cold and desiccation resistance have been under strong directional selection in the near past so that alleles have been fixed in the tropical species. With the decay hypothesis, where loci involved in desiccation and cold resistance would be non-functional we would expect no inbreeding depression in tropical species for cold and desiccation resistance. Our results fail to support this hypothesis since no difference in levels of inbreeding depression between tropical and widespread species were observed for the two traits (Fig. 1d,e,f). According to the second hypothesis we would expect more inbreeding depression in tropical species for desiccation and cold resistance. This is because selection theory and empirical evidence suggests that inbreeding depression will be more severe for traits shaped by (past) directional selection [20]–[22]. Our data do also not support the hypothesis that low additive genetic variance in tropical species can be explained by depletion of additive genetic variance induced by directional selection, since we do not see a difference in inbreeding depression in cold and desiccation resistance between tropical and widespread species (Fig. 1d,e,f).

Our results show that inbreeding depression was strongest for the trait egg-to-adult viability and that tropical species on average tended to suffer relatively more from inbreeding depression in egg-to-adult viability and developmental time. If we assume that the level of inbreeding depression is more severe in traits under directional selection for which there is strong empirical evidence [20]–[22], this may indicate that 1) among the six traits we investigated, egg-to-adult viability has been under strongest directional selection in the past, and 2) egg-to-adult viability on average is under stronger selection in tropically restricted species compared to widespread species.

Effective population sizes are expected to influence the effect of inbreeding, since at low effective population sizes more slightly deleterious variants may be segregating due to strong drift and weak selection. One might speculate that tropical species in general would have low effective population sizes due to small distribution ranges, and therefore predicted to show stronger effects of inbreeding. On the other hand historical inbreeding might also purge deleterious alleles and thus reduce deleterious consequences of inbreeding [23]. The net outcome of effects of effective population size on fitness consequences of consecutive full sib mating is therefore difficult to predict. We have no information on effective population sizes in the investigated species but their high fertility and observations done during sampling of the populations suggest that census sizes are very high for all species. High and similar heritabilities for morphological traits in the investigated species provide indirect evidence for this claim [10], [11].

Inbreeding depression is commonly observed to be more severe for traits closely associated with fitness. Thus, the observation that egg to adult viability is the traits mostly affected by inbreeding support previous results; our data provide evidence from multiple species that there is a fundamental difference between life history and stress response traits with respect to the underlying genetic variation that gives rise to inbreeding depression.

The environment is expected to change dramatically in the future. Temperatures will increase and be more variable and many other climatic variables will change [24]. A central question in regard to this is whether animals and plants are able to respond to these changes through evolutionary and plastic adaptations. As discussed above, tropical species have been hypothesized to be especially prone to population reductions and extinctions due to phylogenetic constraints, lower basal resistance levels and lower adaptive potential in ecological key traits [9]–[11], [25]. For life history traits our results suggest that this will be reinforced by more inbreeding depression in tropical species. The variation in impact of inbreeding between species and lineages within and between species groups observed in this study however reveals the complexity of inbreeding effects; to fully understand the effect of species group on levels of inbreeding depression we suggest testing more species in multiple and ecologically relevant environments.

## Supporting Information

Table S1 **Mean trait values of control (‘c’) and inbred (‘i’) flies (with standard errors in parentheses) for each sex, species and trait observed in this study.** ‘N’ equals the number of inbred lines tested (in parentheses the number of lines available after the 4 generations of full sub mating). N equals 3 for all traits in both males and females in the control lines. The species are: *D. bipectinata* (bipect), *D. birchii* (birc), *D. bunnanda* (bunn), *D. hydei* (hydei), *D. melanogaster* (mel), *D. pseudoananassae* (ps), *D. repleta* (rep), *D. serrata* (ser), *D. simulans* (sim) and *D. sulfurigaster* (sulf). Symbols: ‘T’ = ‘tropical’ and ‘W’ = ‘widespread’.
(DOCX)

## References

[1] Hoffmann AA, Parsons PA (1997) Extreme environmental change and evolution. Cambridge: Cambridge University Press. xii,259p p.

[2] Kristensen TN, Sorensen AC (2005) Inbreeding - lessons from animal breeding, evolutionary biology and conservation genetics. Animal Science 80: 121–133.

[3] Angilletta MJ (2009) Thermal adaptation : a theoretical and empirical synthesis. Oxford: Oxford University Press. xii, 289 p., 282 p. of plates p.

[4] Chown SL, Terblanche JS (2007) Physiological diversity in insects: Ecological and evolutionary contexts. In: Simpson SJ, editor. Advances in Insect Physiology, Vol 33. pp. 50–152. 10.1016/S0065-2806(06)33002-0 PMC2638997 19212462

[5] Ghalambor CK, Huey RB, Martin PR, Tewksbury JJ, Wang G (2006) Are mountain passes higher in the tropics? Janzen's hypothesis revisited. Integrative and Comparative Biology 46: 5–17. 21672718 10.1093/icb/icj003

[6] Kellermann V, Loeschcke V, Hoffmann AA, Kristensen TN, Fløjgaard C, et al (2012) Phylogenetic constraints in key functional traits behind species' climate niches: pattersn of desiccation and cold tolerance across 95 Drosophila species. Evolution In press. 10.1111/j.1558-5646.2012.01685.x 23106704

[7] Hoffmann AA, Watson M (1993) Geographical Variation in the Acclimation Responses of Drosophila to Temperature Extremes. American Naturalist 142: S93–S113. 10.1086/285525 19425954

[8] Overgaard J, Kristensen TN, Mitchell KA, Hoffmann AA (2011) Thermal Tolerance in Widespread and Tropical Drosophila Species: Does Phenotypic Plasticity Increase with Latitude? American Naturalist 178: S80–S96. 10.1086/661780 21956094

[9] Kellermann V, Overgaard J, Hoffmann AA, Flojgaard C, Svenning J-C, et al (2012) Upper thermal limits of Drosophila are linked to species distributions and strongly constrained phylogenetically. Proceedings of the National Academy of Sciences of the United States of America 109: 16228–16233. 22988106 10.1073/pnas.1207553109 PMC3479592

[10] Hoffmann AA, Hallas RJ, Dean JA, Schiffer M (2003) Low potential for climatic stress adaptation in a rainforest Drosophila species. Science 301: 100–102. 12843394 10.1126/science.1084296

[11] Kellermann V, van Heerwaarden B, Sgro CM, Hoffmann AA (2009) Fundamental Evolutionary Limits in Ecological Traits Drive Drosophila Species Distributions. Science 325: 1244–1246. 19729654 10.1126/science.1175443

[12] van Heerwaarden B, Kellermann V, Schiffer M, Blacket M, Sgro CM, et al (2009) Testing evolutionary hypotheses about species borders: patterns of genetic variation towards the southern borders of two rainforest Drosophila and a related habitat generalist. Proceedings of the Royal Society B-Biological Sciences 276: 1517–1526. 10.1098/rspb.2008.1288 PMC2677230 19324823

[13] Nei M, Maruyama T, Chakraborty R (1975) BOTTLENECK EFFECT AND GENETIC-VARIABILITY IN POPULATIONS. Evolution 29: 1–10. 28563291 10.1111/j.1558-5646.1975.tb00807.x

[14] Kristensen TN, Loeschcke V, Bilde T, Hoffmann AA, Sgro C, et al (2011) No Inbreeding Depression for Low Temperature Developmental Acclimation across Multiple Drosophila Species. Evolution 65: 3195–3201. 22023585 10.1111/j.1558-5646.2011.01359.x

[15] Overgaard J, Kristensen TN, Sorensen JG (2012) Validity of Thermal Ramping Assays Used to Assess Thermal Tolerance in Arthropods. Plos One 7. 10.1371/journal.pone.0032758 PMC3302897 22427876

[16] Lande R, Schemske DW (1985) The Evolution of Self-Fertilization and Inbreeding Depression in Plants .1. Genetic Models. Evolution 39: 24–40. 28563655 10.1111/j.1558-5646.1985.tb04077.x

[17] Reed DH, Lowe EH, Briscoe DA, Frankham R (2003) Inbreeding and extinction: Effects of rate of inbreeding. Conservation Genetics 4: 405–410.

[18] Wang JL, Hill WG, Charlesworth D, Charlesworth B (1999) Dynamics of inbreeding depression due to deleterious mutations in small populations: mutation parameters and inbreeding rate. Genetical Research 74: 165–178. 10584559 10.1017/s0016672399003900

[19] Hoffmann AA (2010) Physiological climatic limits in Drosophila: patterns and implications. Journal of Experimental Biology 213: 870–880. 20190112 10.1242/jeb.037630

[20] DeRose MA, Roff DA (1999) A comparison of inbreeding depression in life-history and morphological traits in animals. Evolution 53: 1288–1292. 28565531 10.1111/j.1558-5646.1999.tb04541.x

[21] Falconer DS, Mackay TFC (1996) Introduction to quantitative genetics: Longman Group Limited. xv + 464 p.

[22] Lynch M, Walsh B (1998) Genetics and analysis of quantitative traits: Sinauer Associates, Inc. {a}, 108 North Main Street, Sunderland, Massachusetts 01375, USA. xvi+980p p.

[23] Swindell WR, Bouzat JL (2006) Reduced inbreeding depression due to historical inbreeding in Drosophila melanogaster: evidence for purging. Journal of Evolutionary Biology 19: 1257–1264. 16780526 10.1111/j.1420-9101.2005.01074.x

[24] Solomon S, Intergovernmental Panel on Climate Change., Intergovernmental Panel on Climate Change. Working Group I. (2007) Climate change 2007 : the physical science basis : contribution of Working Group I to the Fourth Assessment Report of the Intergovernmental Panel on Climate Change. Cambridge; New York: Cambridge University Press. viii, 996 p. p.

[25] Deutsch CA, Tewksbury JJ, Huey RB, Sheldon KS, Ghalambor CK, et al (2008) Impacts of climate warming on terrestrial ectotherms across latitude. Proceedings of the National Academy of Sciences of the United States of America 105: 6668–6672. 18458348 10.1073/pnas.0709472105 PMC2373333

