# Supplementary material for: A Comparison of Inbreeding Depression in Tropical and Widespread Drosophila Species
Source: PLoS One. 2013 Feb 27;8(2):e51176. doi: 10.1371/journal.pone.0051176 (PMC3584098; doi:10.1371/journal.pone.0051176)
Supplement: Table S1 — Mean trait values of control (‘c’) and inbred (‘i’) flies (with standard errors in parentheses) for each sex, species and trait observed in this study. ‘N’ equals the number of inbred lines tested (in parentheses the number of lines available after the 4 generations of full sub mating). N equals 3 for all traits in both males and females in the control lines. The species are: D. bipectinata (bipect), D. birchii (birc), D. bunnanda (bunn), D. hydei (hydei), D. melanogaster (mel), D. pseudoananassae (ps), D. repleta (rep), D. serrata (ser), D. simulans (sim) and D. sulfurigaster (sulf). Symbols: ‘T’ = ‘tropical’ and ‘W’ = ‘widespread’. (DOCX) [file pone.0051176.s001.docx]

| Species | Sex | Developmental time (days ±SE) | Egg-to-adult viability (% ±SE) | CTmin (degree ±SE) | Chill coma recovery (sec ±SE) | Desiccation (hours ±SE) | Heat knock  down (sec ±SE) |
| --- | --- | --- | --- | --- | --- | --- | --- |
| bipect-T | Females-c  Females-i  N | 12.4 (0.05)  13.2 (0.18)  7 | 0.81 (0.05)  0.40 (0.07)  7 | 8.56 (0.18)  8.75 (0.17)  7 | 4878 (227)  5787 (574)  5 | 3.70 (0.56)  4.02 (0.36)  3 | 1133 (45)  940 (36)  2 |
|  | Males-c  Males-i  N | 12.9 (0.07)  13.6 (0.19)  7 | 0.80 (0.08)  0.43 (0.07)  7 | 8.22 (0.42)  8.94 (0.15)  7 | 5221 (329)  5783 (133)  5 | 3.76 (0.39)  2.80 (0.21)  3 | 1066 (14)  994 (41)  3 |
| birc-T | Females-c  Females-i  N | 15.3 (0.19)  13.3 (0.19)  4 | 0.56 (0.05)  0.43 (0.08)  4 | 7.77 (0.16)  8.27 (0.32)  4 | 3169 (93)  2991 (266)  4 | 9.86 (0.28)  8.49 (1.01)  4 | 961 (61)  939 (57)  4 |
|  | Males-c  Males-i  N | 15.7 (0.06)  16.9 (0.16)  4 | 0.56 (0.08)  0.44 (0.08)  4 | 7.42 (0.06)  7.81 (0.37)  4 | 3229 (73)  2913 (190)  4 | 7.26 (1.96)  6.12 (0.52)  4 | 912 (48)  962 (37)  4 |
| bunn-T | Females-c  Females-i  N | 16.9 (0.16)  17.4 (0.18)  6 | 0.56 (0.07)  0.25 (0.07)  6 | 7.34 (0.42)  7.79 (0.28)  5 | 2530 (55)  2632 (211)  5 | 11.50 (0.67)  10.77 (0.53)  5 | 684 (105)  847 (70)  5 |
|  | Males-c  Males-i  N | 17.4 (0.09)  17.9 (0.11)  6 | 0.58 (0.07)  0.27 (0.09)  6 | 7.52 (0.15)  7.63 (0.10)  5 | 2589 (86)  2972 (232)  5 | 8.50 (0.10)  9.13 (0.70)  5 | 778 (33)  699 (58)  4 |
| ps-T | Females-c  Females-i  N | 12.2 (0.06)  13.2 (0.19)  10 | 0.69 (0.03)  0.40 (0.06)  10 | 8.35 (0.13)  8.54 (0.34)  9 | 4613 (263)  4131 (94)  8 | 6.66 (1.68)  7.09 (0.52)  8 | 927 (80)  941 (136)  4 |
|  | Males-c  Males-i  N | 12.7 (0.04)  13.4 (0.17)  10 | 0.81 (0.05)  0.42 (0.07)  10 | 8.12 (0.21)  8.59 (0.27)  9 | 6170 (343)  4838 (212)  8 | 6.54 (1.58)  5.67 (0.45)  8 | 921 (90)  846 (151)  4 |
| sulf-T | Females-c  Females-i  N | 12.1 (0.06)  13.0 (0.23)  10 | 0.94 (0.05)  0.48 (0.07)  10 | 9.24 (0.15)  9.04 (0.18)  10 | 2874 (198)  3250 (103)  10 | 11.29 (0.63)  11.05 (0.40)  10 | 939 (164)  950 (81)  3 |
|  | Males-c  Males-i  N | 12.1 (0.05)  12.9 (0.18)  10 | 0.93 (0.03)  0.47 (0.06)  10 | 8.87 (0.14)  9.11 (0.20)  10 | 3348 (250)  3642 (127)  10 | 11.38 (0.65)  9.39 (0.43)  10 | 861 (127)  868 (193)  4 |
| hydei-W | Females-c  Females-i  N | 18.5 (0.01)  19.5 (0.10)  4 | 0.70 (0.11)  0.21 (0.06)  4 | 2.85 (0.14)  2.95 (0.45)  7 | 1625 (100)  1559 (125)  7 | 14.86 (1.43)  13.30 (0.66)  6 | 2147 (82)  2246 (62)  7 |
|  | Males-c  Males-i  N | 19.1 (0.02)  19.7 (0.24)  4 | 0.74 (0.09)  0.21 (0.03)  4 | 2.75 (0.18)  3.83 (0.34)  6 | 1511 (146)  1634 (95)  7 | 20.46 (0.69)  19.26 (0.67)  6 | 2261 (75)  2104 (129)  6 |
| mel-W | Females-c  Females-i  N | 13.3 (0.08)  13.6 (0.23)  6 | 0.84 (0.03)  0.33 (0.07)  6 | 3.30 (0.13)  3.69 (0.17)  6 | 1760 (54)  2167 (110)  6 | 16.68 (0.19)  15.38 (0.44)  5 | 1941 (49)  2192 (59)  3 |
|  | Males-c  Males-i  N | 13.7 (0.06)  14.0 (0.21)  6 | 0.86 (0.02)  0.37 (0.09)  6 | 3.18 (0.25)  3.29 (0.14)  6 | 1662 (112)  1989 (108)  6 | 14.30 (1.22)  13.95 (0.76)  4 | 2509 (120)  2266 (49)  4 |
| rep-W | Females-c  Females-i  N | 15.8 (0.10)  16.3 (0.22)  10 | 0.50 (0.13)  0.59 (0.05)  10 | 6.27 (0.29)  5.82 (0.16)  10 | 1712 (76)  2056 (129)  10 | 14.46 (1.42)  14.75 (0.93)  10 | 1105 (30)  1121 (65)  8 |
|  | Males-c  Males-i  N | 16.1 (0.13)  16.5 (0.19)  10 | 0.47 (0.12)  0.56 (0.07)  10 | 5.07 (0.23)  5.70 (0.19)  10 | 1711 (19)  1900 (142)  10 | 14.49 (1.03)  15.87 (0.88)  10 | 1212 (66)  1066 (57)  9 |
| ser-W | Females-c  Females-i  N | 16.3 (0.15)  16.3 (0.16)  10 | 0.35 (0.05)  0.56 (0.05)  10 | 5.08 (0.08)  5.68 (0.20)  10 | 2033 (31)  2107 (100)  10 | 18.10 (0.49)  15.01 (1.34)  9 | 1319 (78)  1378 (467)  5 |
|  | Males-c  Males-i  N | 16.7 (0.13)  16.9 (0.14)  10 | 0.42 (0.11)  0.54 (0.06)  10 | 5.09 (0.24)  5.18 (0.28)  10 | 1918 (25)  2418 (136)  9 | 10.63 (0.52)  10.22 (1.05)  9 | 1367 (67)  1232 (269)  9 |
| sim-W | Females-c  Females-i  N | 12.0 (0.12)  12.3 (0.18)  3 | 0.82 (0.06)  0.58 (0.19)  3 | 4.38 (0.42)  4.50 (0.60)  3 | 2275 (122)  2100 (259)  3 | 10.29 (0.55)  9.29 (1.65)  3 | 1290 (33)  1475 (52)  2 |
|  | Males-c  Males-i  N | 12.5 (0.02)  12.7 (0.21)  3 | 0.92 (0.02)  0.63 (0.18)  3 | 4.16 (0.24)  4.19 (0.36)  3 | 2092 (125)  2154 (239)  3 | 8.39 (0.40)  6.73 (0.38)  3 | 1451 (56)  1423 (50)  3 |
